# Comparative Evaluation of Herbal Versus Non-herbal Dentifrice in Maintaining Oral Health of Young Adults

**DOI:** 10.7759/cureus.65331

**Published:** 2024-07-25

**Authors:** Shivlingesh KK, Chanchal Gangwar, Swati Sharma, Rupali Kalsi, Garima Asthana, Vineeta Gupta

**Affiliations:** 1 Department of Public Health Dentistry, Institute of Dental Sciences, Bareilly, IND; 2 Department of Public Health Dentistry, School of Dental Sciences, Sharda University, Noida, IND; 3 Department of Periodontology, Government Institute of Medical Sciences, Noida, IND; 4 Department of Periodontics, Manav Rachna Dental College, Faridabad, IND; 5 Department of Periodontics, Government Dental College, Raipur, IND

**Keywords:** oral health, synthetic toothpaste, plaque control, herbal toothpaste, gingivitis

## Abstract

Background: Dental plaque may be attributed as a precursor to various oral health problems like dental caries, periodontal disease, halitosis, etc. With an ever-increasing awareness about the adverse effects of chemical formulations, emphasis is now being laid on the usage of herbal ingredients, as they are safer for long-term use in addition to their medicinal benefits.

Aim: The present study aims to assess the anti-plaque and anti-inflammatory efficacy of herbal toothpaste compared to synthetic toothpaste among 20-40-year-old patients in Bareilly, Uttar Pradesh.

Methodology: In this study, 130 subjects aged between 20 and 40 years with poor oral hygiene status and signs of gingival inflammation corresponding with chronic marginal gingivitis were selected. Subjects in Group 1 were prescribed herbal toothpaste (KUDOS Ayurveda), whereas patients in Group 2 were administered non-herbal toothpaste. Clinical parameters of gingival health specifically gingival index (GI), patient hygiene performance (PHP), and approximate plaque index (API) were recorded at baseline, 14 days, 28 days, and 42 days. Chi-square test, student-independent t-test, and paired t-test were performed to find significant differences in various variables between the two groups at different follow-up visits.

Results: At the end of the study, a significant reduction in GI and API scores was obtained, along with increased PHP scores. The p-value was set at 0.05, and the power of the study was set at 0.95. There was a statistically significant reduction (p < 0.05) in GI and API and an improvement in PHP scores at various time intervals in Group 1 as compared to Group 2.

Conclusion: Regular application of herbal extract dentifrices for 42 days provided a significant reduction of dental plaque and improvement in overall gingival health without any adverse effects. This instilled motivation in the patients to uphold proper oral hygiene. Hence, herbal dentifrices could be prescribed as an adjunct to periodontal therapy in the maintenance phase.

## Introduction

The oral cavity is a reflector that gives an insight into the overall health conditions of our body. Various local and systemic factors are responsible for poor oral health thereby causing dental caries, periodontal diseases, halitosis, etc., but dental plaque may be attributed as a precursor of the abovementioned conditions. Gingivitis caused by plaque is the second most prevalent oral illness after dental caries and affects about 75% of the global population [1]. Therefore, various plaque control methods including mechanical and chemical plaque control regimes have been employed to restrict the deposition of dental plaque in the oral cavity. However, the self-motivation of an individual for regular plaque removal has been considered paramount in preventing and controlling gingivitis [2]. Chlorhexidine and triclosan are the most commonly used chemical formulations for plaque control, but their prolonged usage stains the teeth and alter taste sensation [3]. Herbal toothpastes are indigenous formulations with natural ingredients having antimicrobial and anti-inflammatory properties. Neem (*Azadirachta indica*), Babool (*Acacia arabica*), haldi (*Curcuma longa*), etc., are safer for prolonged use with minimal side effects in addition to their medicinal benefits. Neem, among other herbal ingredients, possesses anti-inflammatory and anti-carious properties [4]. Allergic reactions mimicking plasma cell gingivitis are reported in a few case reports with the use of herbal dentifrices [5]. Therefore, the present study aims to assess the anti-plaque and anti-inflammatory efficacy of herbal (KUDOS Neem Clove toothpaste) to non-herbal toothpaste among 20-40-year old patients of Bareilly City, Uttar Pradesh.

## Materials and methods

After obtaining clearance from the Institutional Ethics Committee of the Institute of Dental Sciences (IEC No. IEC/131/2021) and signing informed consent from patients, data was collected from the Out Patient Department (OPD) of Public Health Dentistry, Institute of Dental Sciences, Bareilly, Uttar Pradesh. The sample size was calculated using the formula: n = (Z_α/2_​+Z_β_​​)^2^/Δ/σ, where n is the sample size per group; Z_α/2_ is the critical value of the normal distribution at α/2 (for a 95% confidence level; α = 0.05 Zα/2 = 1.96); Z_β​_ is the critical value of the normal distribution for the power 1−β (for a power of 0.95, β = 0.05, and Zβ = 1.645); Δ is the expected difference between the means of the two groups; and σ is the standard deviation of the outcome variable.

The minimum sample size calculated according to the formula was 118 and was increased to 130 to compensate for the loss of subjects during subsequent follow-ups. The p-value was set at 0.05, and the power of the study was set at 0.95. A total of 130 systematically healthy subjects aged between 20 and 40 years suffering from plaque-induced gingivitis with at least 20 teeth in their oral cavity were recruited for the study as per the AAP 1999 classification [6]. Clinical parameters of gingival health such as gingival index (GI), patient hygiene performance (PHP), and approximate plaque index (API) were recorded at baseline, 14 days, 28 days, and 42 days.

GI scores were used to assess bleeding on probing, and the obtained scores described the type of gingival inflammation [7]. PHP index was used to assess the oral hygiene maintenance by the subjects in which the disclosing agent was applied to the index teeth of the subjects for 30 seconds. The oral debris was stained dark pink [8].

API was used to assess the presence or absence of plaque in the interproximal areas after the application of the disclosing agent [7]. The interpretations were based on the percentage of area covered by the plaque in the interproximal space.

Exclusion criteria were patients with fixed orthodontic appliances, advanced periodontal inflammation, a history of antimicrobial or anti-inflammatory medication, pregnant or lactating females, and individuals with systemic diseases. Noncompliant subjects were also excluded from the study. A total of 130 selected participants were randomly allocated into two study groups using computer-generated random allocation software with 65 subjects in each group. All toothpastes were colored black to ensure blinding. Active ingredients of non-herbal toothpaste used for study were calcium carbonate, sorbitol, sodium lauryl sulfate, silica, titanium dioxide, titanium dioxide, sodium silicate, flavor carrageenan, sodium monofluorophosphate, sodium bicarbonate, benzyl alcohol, sodium saccharin, and triclosan, in aqueous base, whereas the contents of herbal toothpaste used were *Azadirachta indica, Apium leptophyllum, Zanthoxylum armatum, Acacia nilotica, Phyllanthus emblica, Emblica officinalis, Terminalia chebula, Terminalia befenca, Vitex negundo, Embelia ribes, Aloe barbadensis, Mentha vindis Linn, Ocimum sanctum, Rubia cordifolia, Quercus infectoria, Mojisari, Citrus limonum, Gulab, and Syzygium aromaticum.*

Group 1 subjects were prescribed KUDOS Neem Clove toothpaste (KUDOS Laboratories, Haryana, India). Group 2 participants were prescribed non-herbal toothpaste. They were instructed to use respective toothpaste twice daily with modified bass technique for 42 days. A modified case-history proforma including demographic details, the GI, PHP, and API was used to record data obtained from the subjects by a precalibrated examiner. Subjects were assessed at baseline, 14 days, 28 days, and 42 days for the clinical parameters. The obtained data was then statistically analyzed.

Statistical analysis

Statistical analysis was done by IBM SPSS Statistics for Windows, Version 20 (Released 2011; IBM Corp., Armonk, New York, United States). The chi-square was performed for an intergroup association between various variables. Student-independent t-tests and paired t-tests were performed for intragroup and intergroup comparisons, respectively, at follow-up visits. A p-value ≥ 0.05 was considered statistically significant.

## Results

This descriptive analysis outlines the gender distribution within each group. A total of 130 subjects were equally distributed into two groups. Group 1 (herbal toothpaste group) consisted of 33 males (50.8%) and 32 females (49.2%), while Group 2 (non-herbal toothpaste group) consisted of 34 males (52.3%) and 31 females (47.7%) (Table 1).

**Table 1:** Demographic details of the selected population

Variables	Males (n%)	Females (n%)	Total (n%)	Age in years (mean ± SD)
Group 1 (herbal toothpaste group)	33 (50.8)	32 (49.2)	65 (100)	26.55 ± 2.49
Group 2 (non-herbal toothpaste group)	34 (52.3)	31 (47.7)	65 (100)	27.12 ± 2.18

The GI was used to assess the prevalence and severity of gingival inflammation. Group 1 showed significant improvement in the GI scores at day 14 (2.44 ± 0.32), day 28 (1.25 ± 0.19), and day 42 (0.59 ± 0.33) compared to Group 2, which had scores of 2.55 ± 0.31 at day 14, 1.34 ± 0.22 at day 28, and 0.71 ± 0.35 at day 42 (Table 2).

**Table 2:** Intergroup comparison between various periodontal parameters

Variables	Examination days	Group 1 (mean ± SD)	Group 2 (mean ± SD)	p-value
Gingival index (GI)	Baseline	2.63 ± 0.31	2.65 ± 0.29	0.70
	Day 14	2.44 ± 0.32	2.55 ± 0.31	0.05
	Day 28	1.25 ± 0.19	1.34 ± 0.22	0.01
	Day 42	0.59 ± 0.33	0.71 ± 0.35	0.05
Approximal plaque index (API)	Baseline	51.82 ± 7.85	51.98 ± 7.76	0.90
	Day 14	34.94 ± 1.73	35.62 ± 1.78	0.03
	Day 28	31.13 ± 2.73	32.16 ± 2.69	0.03
	Day 42	24.95 ± 1.21	25.43 ± 1.23	0.03
Patient hygiene performance index (PHP)	Baseline	3.83 ± 0.54	3.73 ± 0.63	0.36
	Day 14	3.38 ± 0.52	3.62 ± 0.56	0.01
	Day 28	2.75 ± 0.41	2.98 ± 0.45	0.01
	Day 42	2.07 ± 0.37	2.21 ± 0.38	0.03

The API, which assesses inflammation in interproximal areas by measuring bleeding on probing, also showed significant improvement (p ≤ 0.05) in Group 1. The API scores were 34.94 ± 1.73 at day 14, 31.13 ± 2.73 at day 28, and 24.95 ± 1.21 at day 42. These improvements indicate better gingival health in Group 1 (Table 3).

**Table 3:** Intragroup comparison in Group 1 between the periodontal parameters

Variables	Group 1 (mean ± SD)		Mean difference	p-value
	Baseline	Day 42		
Gingival index (GI)	2.63 ± 0.31	0.59 ± 0.33	2.04	0.001
Approximal plaque index (API)	51.82 ± 7.85	24.95 ± 1.21	26.87	0.001
Patient hygiene performance index (PHP)	3.83 ± 0.54	2.07 ± 0.37	1.76	0.001

The PHP index, which allows patients to assess their oral hygiene maintenance, showed that Group 2 had higher scores at day 14 (3.38 ± 0.52), day 28 (2.75 ± 0.41), and day 42 (2.07 ± 0.37). However, Group 1 also displayed significant improvements compared to baseline scores. These results highlight the medicinal benefits of herbal products and increased patient motivation to maintain oral hygiene (Table 4).

**Table 4:** Intragroup comparison in Group 2 between the periodontal parameters

Variables	Group 2 (mean ± SD)		Mean difference	p-value
	Baseline	Day 42		
Gingival index (GI)	2.65 ± 0.29	0.71 ± 0.35	1.94	0.001
Approximal plaque index (API)	51.98 ± 7.76	25.43 ± 1.23	26.55	0.001
Patient hygiene performance index (PHP)	3.73 ± 0.63	2.21 ± 0.38	1.52	0.001

All parameters were analyzed within each group using a paired t-test. The results revealed highly significant improvements in both groups. In Group 1, the mean differences were 2.04 for GI, 26.87 for API, and 1.76 for PHP, all statistically highly significant. In Group 2, the mean differences were 1.94 for GI, 26.55 for API, and 1.52 for PHP, also highly significant. The significant improvement in all the parameters in both groups can be attributed to similar oral hygiene instructions, motivation, and demonstration of proper brushing technique given to all the subjects in both groups. Despite the significant results in both groups, Group 1 showed better clinical improvement in oral health, indicating the added medicinal benefits of indigenous ingredients of the herbal toothpaste.

## Discussion

Failure to achieve an optimum level of oral hygiene that should be commensurate with periodontal health led to an increased interest in the incorporation of indigenous ingredients into the current formulation of dentifrices, especially in a country like India, which holds a golden history of Ayurveda. Native herbal medicines are gaining special interest in the dental field for preventing and curing dental diseases. Data about the efficacy of these products is sparse, and it is therefore imperative that clinical trials verify the efficacy of new products [2].

The primary goal of the present study was to compare the anti-plaque and anti-inflammatory efficacy of herbal and non-herbal dentifrices that are commercially available. In this study, the main herbal ingredients used in dentifrice are neem and lavang (clove), whose medicinal effects have been invariably used in medical treatment. Neem has displayed antibacterial, antioxidant, anti-inflammatory, host immune modulation, and anti-plaque effects with low or no toxicity. Its antioxidant effect acts against *S. mutans*, thereby enhancing the host immune potential in combating periodontal disease [9]. At the same time, lavang (clove) also offers a variety of medicinal properties like antioxidant, antiseptic, antibacterial, analgesic, and bacteriostatic effects, which help combat microbial infections and reduce inflammation [10]. In this study, a statistically significant reduction in the GI and API scores was observed in Group 1 between baseline and day 42. These results were from a study where commercial fluoride-triclosan dentifrice was compared to a herbal dentifrice containing green tea extracts. The herbal dentifrice group showed a marked reduction in gingival inflammation and other periodontal parameters, thereby concluding that green tea dentifrice may serve as an adjunct to periodontal therapy [11]. Another randomized controlled trial compared *Carica papaya *leaf extract dentifrice to placebo and found a marked reduction in gingival bleeding and inflammation in the test group. Hence, herbal extract could be a viable alternative to synthetic toothpaste in daily use [12].

On day 42, Group 1 showed a significant improvement in the PHP index compared to the baseline, indicating the subjects' motivation to maintain good oral hygiene. Another study, which compared the use of *S. baicalensis* extract-containing dentifrice to a placebo, found an improvement in the gingival score and plaque score, concluding that the test dentifrice was more effective in reducing inflammation. Hence, it can be used safely daily [13]. However, a few studies concluded that there was no significant difference in the efficacy of both dentifrices. A randomized controlled trial was conducted comparing Parodontax toothpaste to fluoridated toothpaste. In this study, the authors concluded that no added clinical advantage was offered by Parodontax in comparison to fluoridated toothpaste. Although it did reduce plaque deposition and gingivitis [14]. Another study compared Colgate Total toothpaste to placebo and derived a similar conclusion as the previous study, showing no difference between the test dentifrice and the positive control dentifrice [15,16]. In an in vitro study, the maximum zone of inhibition was observed when culturing the sample treated with herbal toothpaste as compared to chemical toothpaste [17-19]. This study was also conducted to test the efficacy of herbal toothpaste in comparison to non-herbal dentifrice. Within the limitations of the study, the authors concluded that herbal toothpastes showed better results in terms of reductions in plaque deposition and gingival inflammation. Also, the subjects enrolled in the study were more motivated and displayed improved oral hygiene maintenance without reporting any adverse events related to the use of herbal dentifrices. These results have been proven in various studies and literature reviews [20-22]. 

Our study on the efficacy of herbal versus non-herbal dentifrices has limitations, including a small sample size and a short 42-day follow-up period, which may not capture long-term effects and side effects. The reliance on self-reported oral hygiene practices could introduce bias, and the single-institution setting limits generalizability. Additionally, the diverse formulations of dentifrices mean the results may not apply to all products. Future research should involve larger, more diverse populations, longer follow-up periods, and multicenter trials to validate these findings and ensure broader applicability.

## Conclusions

Based on improved outcomes in both groups, it can be concluded that it is worthy to give directives about good oral hygiene practices to the patients. Better clinical improvement in oral health with herbal toothpaste can be attributed to the additional medicinal benefits of Indigenous ingredients of the dentifrice. Though no allergic reaction was reported in any of the subjects, longitudinal studies are still required to establish the beneficial effect and safety of herbal dentifrices in maintaining oral health in the long term.
